# A deep-learning approach for reconstructing 3D turbulent flows from 2D observation data

**DOI:** 10.1038/s41598-023-29525-9

**Published:** 2023-02-13

**Authors:** Mustafa Z. Yousif, Linqi Yu, Sergio Hoyas, Ricardo Vinuesa, HeeChang Lim

**Affiliations:** 1 School of Mechanical Engineering, Pusan National University, Busandaehak-ro, Busan, 46241 Republic of Korea; 2 Instituto Universitario de Matemática Pura y Aplicada, Universitat Politècnica de València, 46022 Valencia, Spain; 3 FLOW, Engineering Mechanics, KTH Royal Institute of Technology, 10044 Stockholm, Sweden

**Keywords:** Mechanical engineering, Applied physics, Fluid dynamics

## Abstract

Turbulence is a complex phenomenon that has a chaotic nature with multiple spatio-temporal scales, making predictions of turbulent flows a challenging topic. Nowadays, an abundance of high-fidelity databases can be generated by experimental measurements and numerical simulations, but obtaining such accurate data in full-scale applications is currently not possible. This motivates utilising deep learning on subsets of the available data to reduce the required cost of reconstructing the full flow in such full-scale applications. Here, we develop a generative-adversarial-network (GAN)-based model to reconstruct the three-dimensional velocity fields from flow data represented by a cross-plane of unpaired two-dimensional velocity observations. The model could successfully reconstruct the flow fields with accurate flow structures, statistics and spectra. The results indicate that our model can be successfully utilised for reconstructing three-dimensional flows from two-dimensional experimental measurements. Consequently, a remarkable reduction in the complexity of the experimental setup and the storage cost can be achieved.

## Introduction

Turbulence is probably the unresolved problem of classical physics with the most applications in daily life. The drag caused in the first millimeters of the flow surrounding vehicles or inside pipelines is responsible for up to 5% of the $\text{CO}_2$ emissions caused by humanity every year^1^. Increasing our knowledge of wall-bounded flows is thus a first-order priority. However, the highly non-linear and chaotic nature of turbulent flows has been a great challenge for centuries. Furthermore, the analytical solution of the equations describing the flow around an object, the Navier–Stokes equations, is still impossible today for almost all practical purposes^2^.

With the development of computational power and experimental tools, an accurate description of various types of turbulent flows can be achieved. Different strategies have been proposed when predicting turbulence, namely, the Reynolds-averaged Navier–Stokes (RANS) equations, where all scales are modeled; the large-eddy simulation (LES), where the largest scales are simulated, and the direct numerical simulation (DNS). In DNS, no empirical modeling is needed to account for turbulent effects. The approximations of the solutions of the Navier–Stokes equations are obtained through highly-accurate numerical schemes. The main problematic issue of DNS is its high computational cost since even the smallest scales of turbulence, the Kolmogorov scales^3^, have to be simulated. Hence, this limits DNS to very simple canonical geometries, such as the ones used in this work. However, DNS has the same validity as experiments, and almost any imaginable quantity can be computed.

In the area of experimental fluid dynamics, significant technical advances have been achieved through particle-image velocimetry (PIV)^4^, tomographic-PIV (tomo-PIV)^5^ and four-dimensional time particle-tracking velocimetry (4D-PTV)^6^. However, in both the numerical and experimental approaches, substantial costs are required to describe the physics of the turbulent flows accurately and these costs are proportional to the Reynolds number. This nondimensional parameter is proportional to the size of the problem, its characteristic velocity, and inversely proportional to the fluid kinematic viscosity.

On the other hand, an enormous amount of data can be generated from experimental and numerical studies. Turbulence is now a science that needs, more than ever, new questions more than new data to solve those questions. This motivates developing data-driven methods that can practically utilise the data for addressing various turbulence-related problems. With the recent rapid advances of machine-learning algorithms and the remarkable improvement in the graphic-processing-unit (GPU) capabilities, machine learning has been applied in various fields, including image processing, natural language processing, robotics, weather forecasting, etc. In terms of fluid dynamics, deep-learning algorithms have been effectively applied to tackle a wide range of problems^7–9^, where deep learning is a subset of machine learning in which neural networks with multiple layers are utilised in the model^10^. Unlike conventional linear methods, deep-learning-based techniques can deal with complex non-linear problems. This makes the deep-learning approach a good candidate to be applied for various problems in turbulence, such as turbulence modelling^11–13^, prediction of turbulent flows^14–16^, reduced-order modelling^17,18^, turbulent-flow control^9,19–21^ and non-intrusive sensing^22,23^. The reconstruction of turbulent flows from spatially-limited data using deep-learning-based models has been recently a topic of interest, considering the ability of several deep-learning models to map the flow fields that are represented by spatially-limited or low-resolution data to high-resolution flow fields^23–32^. The results obtained from the cited studies indicate that several deep-learning models have a remarkable potential to map turbulent flow fields with limited spatial distribution to high fidelity flow fields by making use of the available training data. Thus, such deep-learning models possibly can reconstruct the missing regions in the flow fields by compensating them with approximation functions represented by the trainable parameters in the models.

In this study, we present a deep-learning-based method to reconstruct three-dimensional (3D) turbulent flows from two-dimensional (2D) data, in such a way that mimics the reconstruction of 3D turbulent flows from 2D PIV measurements. In contrast with the proposed studies in the literature, which are based on simple approaches such as assumptions of frozen velocity^33–35^ and frozen turbulence via Taylor hypothesis^36^, exploiting homogeneity in the flow^37^ and proper-orthogonal decomposition (POD)^38,39^, we apply a deep-learning-based approach to map unpaired intersected 2D turbulent flow sections to 3D flow fields. We propose a generative-adversarial-network (GAN)-based model, 2D3DGAN, to reconstruct 3D turbulent flows. Unlike the traditional convolutional-neural-networks (CNNs)-based models, GAN-based models^40^ have shown the ability to capture high-frequency data in detail, and remarkable accuracy in terms of image transformation and super-resolution problems^41–44^. In a typical GAN, two networks, namely the generator (*G*) and the discriminator (*D*), compete with each other. Here, *G* generates artificial samples similar to the real ones, whereas *D* distinguishes the artificial samples from the real ones. The goal of the training process is to make *G* generate artificial samples that are difficult to distinguish using *D*. We utilise a combination of 2D and 3D CNNs to build the 2D3DGAN, which can use both the supervised deep-learning method and the adversarial networks, i.e. *G* and *D* networks. We remark that the 2D3DGAN is robust to increasing Reynolds number and the complexity of the flow.

To perform this study, we have selected two different geometries: Turbulent channel flow and the flow around a finite wall-mounted square cylinder. Turbulent channel flows are the simplest complete example of a fully-developed turbulent flow. The flow is between two parallel plates and is driven by a streamwise pressure gradient. Since the seminal simulation of Kim, Moin, and Moser^45^, the complexity of the flows simulated has grown steadily^45–52^. Turbulent channel flow exhibits most of the phenomena needed to understand turbulent flow over surfaces in more general cases. Due to its characteristics, turbulent channel flow is the guinea pig of wall-bounded turbulent flows. Note that each instantaneous flow field in^52^ requires around 400 GB of storage, whereas saving just two-dimensional planes would imply a storage saving of 99.9%. The second flow of interest, the flow around a finite wall-mounted square cylinder^53,54^, is a first representation of a simplified urban flow. This flow represents a different challenge, as the interaction of the flow with the cylinder is extremely complex. In both cases, our method exhibits very good performance, opening the way to use it in more realistic environments.

## Results

### Building 2D3DGAN architecture

The process of training GAN can be expressed as a min-max two-player game with a value function *V*(*D*, *G*) such that:

1 $$\begin{aligned} \begin{aligned} \begin{array}{c} \mathrm min\\ {G} \end{array} ~\begin{array}{c} \mathrm max\\ {D} \end{array} ~V(D,G) = {\mathbb {E}}_{\chi _r \sim P_{\textrm{data}}(\chi _r)} [ \textrm{log} D(\chi _r )] + {\mathbb {E}}_{\zeta \sim P_{\zeta }(\zeta ) } [ \textrm{log} (1-D(G(\zeta )))], \end{aligned} \end{aligned}$$

where $\chi _r$ represents real data and $P_{\textrm{data}} (\chi _r)$ is its distribution. Note that ${\mathbb {E}}$ represents the operation of calculating the average of all the data in the training mini-batch. In the second term of the right-hand side of Eq. (1), $\zeta$ is a random vector used as an input to *G*, whereas $D(\chi _r)$ represents the probability that the data is real and not artificial. The output from *G*, i.e. $G(\zeta )$, is expected to generate data that is similar to the real one, such that the value of $D(G(\zeta ))$ is close to 1. On the other hand, in *D*, $D(\chi _r )$ returns a value close to 1, whereas $D(G(\zeta ))$ returns a value close to 0. Thus, *G* is trained in a direction that minimizes *V*(*D*, *G*), and *D* is trained in a direction that maximizes *V*(*D*, *G*). Additional details are provided in the “Methods” section.

**Figure 1:**
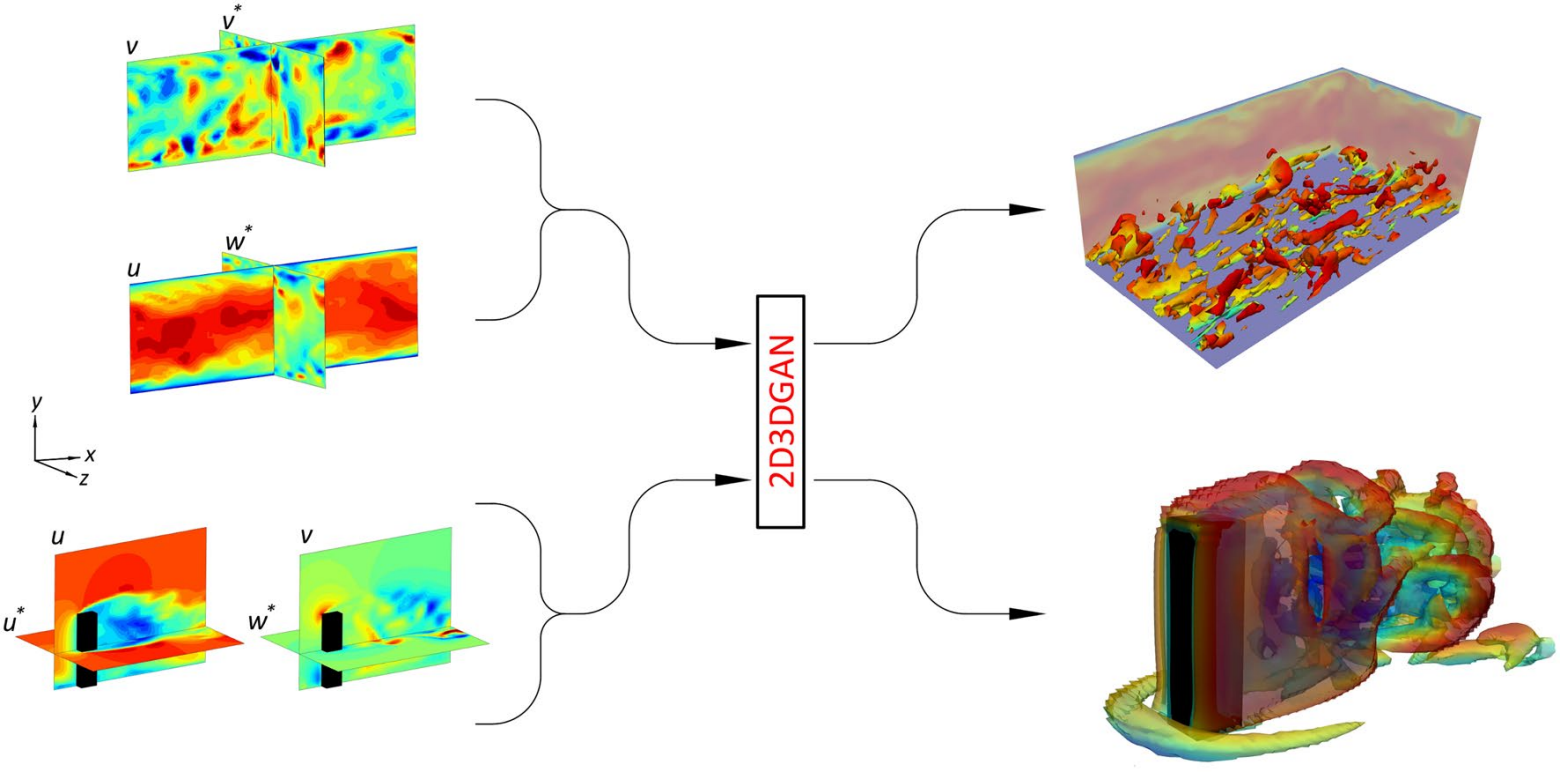
Procedure of reconstructing three-dimensional turbulent flows using the 2D3DGAN. The domain from each simulation^48,55^ contains planar data of the flow obtained from a cross-plane. Note that the flow data of the two planes in the cross plane are entirely unpaired, i.e. the velocity fields in the planes are at different instants. The superscript ‘$*$’ indicates the velocity components that are matched with the velocity data from the observation plane by applying the procedure explained in Eq. (2).

The proposed 2D3DGAN is inspired by the works of Wang et al.^43^ and Yousif et al.^29^, where the input to the network is represented by data that contains limited information about the flow instead of the random vector *z* in Eq. (1). While the models of Refs.^29,43^ are designed to reconstruct 2D high-resolution flow fields from 2D low-resolution data, our 2D3DGAN is designed to map 2D data to 3D flow fields. More details regarding the architecture of the 2D3DGAN can be found in the “Methods” section. As shown in Fig. 1, the input to the 2D3DGAN is data represented by a cross-plane of unpaired intersected 2D flow-observation planes, i.e. each plane contains data of two velocity components, and the data are collected at a period that is different from that of the other plane. Hence, the velocity fields in the planes are at different instants.

In this study, two cases are used to evaluate the performance of the proposed model: a turbulent channel flow at two different friction Reynolds numbers, $Re_\tau =$180 and 500, and the flow around a finite wall-mounted square cylinder with aspect ratio, $AR =$4, at a Reynolds number based on the free stream velocity and the cylinder width *d* of $Re_d = 500$. In both cases, DNS is utilised to generate the flow data.

Let us take the case of turbulent channel flow in Fig. 1 as a demonstration of matching the unpaired data before feeding it to the 2D3DGAN. The data from the two sections are synthetically unpaired such that no instantaneous velocity of each section is found at the same time as the instantaneous velocity in the other section. After that, the flow data of the ($x-y$) section is matched with the data of ($y-z$) section by utilising the square of $L_2$ norm error for the intersection line of the wall-normal velocity such that:

2 $$\begin{aligned} v_{(y-z)}^*,w_{(y-z)}^*= \begin{array}{c} \textrm{argmin}\\ v_{(y-z)}, w_{(y-z)} \end{array} \left( \left\| v_{(x-y)}^y - v_{(y-z)}^y (t) \right\| _2^2 \right) , \end{aligned}$$

where *v* and *w* are the wall-normal and spanwise instantaneous velocity components. Here, $v_{(x-y)}^y$ represents the wall-normal velocity in the $(x-y)$ plane, and $v_{(y-z)}^y (t)$ represents the wall-normal velocity in the $(y-z)$ plane as a function of time, *t*. The superscript ‘$*$’ indicates the matched velocity data using Eq. (2). This procedure mimics the matching of two data sets from two planar PIV experiments, each of them conducted for a different plane. The first plane is the observation plane, which is here represented by the ($x-y$) plane, with velocity components (*u*, *v*), where *u* is the instantaneous streamwise velocity component. The second one is the $(y-z)$ plane with velocity components (*v*, *w*). Note that the selection of the planes is based on maximising the flow information and the 3D label data used for training the model are at the same instants of the observation plane. A similar approach is followed for the case of flow around a finite wall-mounted square cylinder with the observation plane being the central $(x-y)$ plane $(z/d = 0)$. The $(x-z)$ plane at $y/d =2$ is used to provide the matched velocity data with the data from the observation plane.

### Instantaneous flow reconstruction

**Figure 2:**
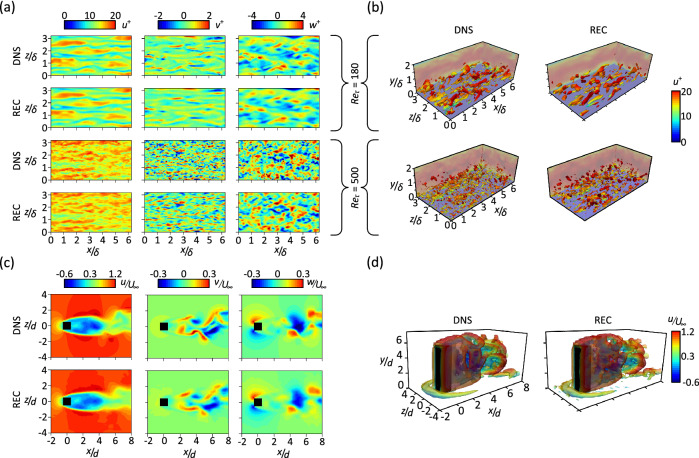
Instantaneous velocity fields and flow structures. (**a**) $(x-z)$ Plane instantaneous velocity fields of the turbulent channel flow case; from left to right: streamwise velocity, wall-normal velocity and spanwise velocity; the planes are located at $y^+ = 16.78$ and 46.95, for the flow at $Re_\tau = 180$ and 500, respectively. (**b**) Isosurfaces of the instantaneous flow structure for the turbulent channel flow case; $Q^+ = 0.006$ and 0.002 for the flow at $Re_\tau =$ 180 and 500, respectively. (**c**) Instantaneous velocity fields for the case of flow around a finite wall-mounted square cylinder at the $(x-z)$ plane, located at $y/d = 3$; from left to right: streamwise velocity, wall-normal velocity and spanwise velocity. (**d**) Isosurfaces of the instantaneous flow structure for the case of flow around a finite wall-mounted square cylinder; $Q (d/U_\infty )^2 = 0.0068$. Note that REC denotes reconstructed field.

First, we examine the ability of the model to reconstruct the 3D instantaneous velocity fields. Figure  2a shows the reconstructed instantaneous velocity fields of the case of turbulent channel flow $(u^+, v^+, w^+ )$ at the $(x-z)$ plane, located at $y^+ = 16.78$ and 46.95, for the flow at $Re_\tau = 180$ and 500, respectively. As can be seen in the figure, the velocity fields are successfully reconstructed by the model with commendable precision even though the $(x-z)$ plane is not introduced to the model during the training process. Note that the superscript ‘$+$’ indicates that the quantity is scaled in inner units using the fluid viscosity, $\nu$ and the friction velocity, $u_\tau$. The flow structure is investigated in Fig. 2b by utilising the *Q*-criterion for vortex identification^56^. As shown in the figure, the reconstructed instantaneous velocity fields reveal a vortical structure $(Q^+)$ that is similar to that obtained from the DNS data, indicating that the model could successfully reproduce the flow fields with high accuracy.

Figure 2c shows the reconstructed instantaneous velocity fields for the case of flow around a finite wall-mounted square cylinder $(u/U_\infty ,v/U_\infty ,w/U_\infty )$ at the (x − z) plane, located at $y/d = 3$, where $U_\infty$ is the free stream velocity. As shown in the figure, all the three velocity fields are in good agreement with the DNS data. Note that the velocity fields along the height of the cylinder show very good agreement with the DNS data even for this region, i.e. near the free end of the cylinder, which is not seen by the model as input during the training process. This indicates that the model can reconstruct the complex three-dimensional turbulent flow around the cylinder within all the regions. Furthermore, the vortical structure obtained from the reconstructed instantaneous velocity fields is generally consistent with the results from DNS as shown in Fig. 2d.

Finally, the accuracy of the reconstruction is examined via the $L_2$-norm relative error:

3 $$\begin{aligned} \epsilon = \frac{1}{J} \sum _{j=1}^J \frac{\left\| {\alpha }_j^{\textrm{DNS}} - {\alpha }_j^{\textrm{REC}}\right\| _2 }{\left\| {\alpha }_j^{\textrm{DNS}}\right\| _2}, \end{aligned}$$

where $\alpha _j^{\textrm{DNS}}$ and $\alpha _j^{\textrm{REC}}$ represent the ground truth (DNS) and the reconstructed instantaneous velocity components, respectively, and *J* represents the number of the test snapshots. As can be observed from Table 1, for the case of turbulent channel flow, no significant increase in the error values is observed in the channel when increasing $Re_{\tau }$ from 180 to 500, indicating the robustness of the model to increasing Reynolds number. Also, for the case of flow around a finite wall-mounted square cylinder, the error values are acceptable as compared to the error values of the turbulent channel, a fact that further supports the ability of the model to reconstruct the velocity fields of complex flows. Note that the errors in *u* are low, while the errors in *v* and *w* are comparatively higher. This is explained by the fact that in these flows the main physics is driven by the streamwise component, which is the main focus of the deep learning model when performing the predictions. It is worth noting here that in GAN-based models, the mapping of high frequency fluctuations in the data is related to the adversarial loss. In other words, in GAN-based models, synthetic data is generated for the high frequency fluctuations. This is the main difference between GAN-based models and traditional CNNs, where the results are usually blurry with few flow details that can be predicted. Also, it is important to note that, despite the deviations in *v* and *w* error values, the main flow features are very well reproduced (as observed in Fig. 2), and as discussed from the statistical and spectral perspectives next.

### Spatial distribution of the flow

**Figure 3:**
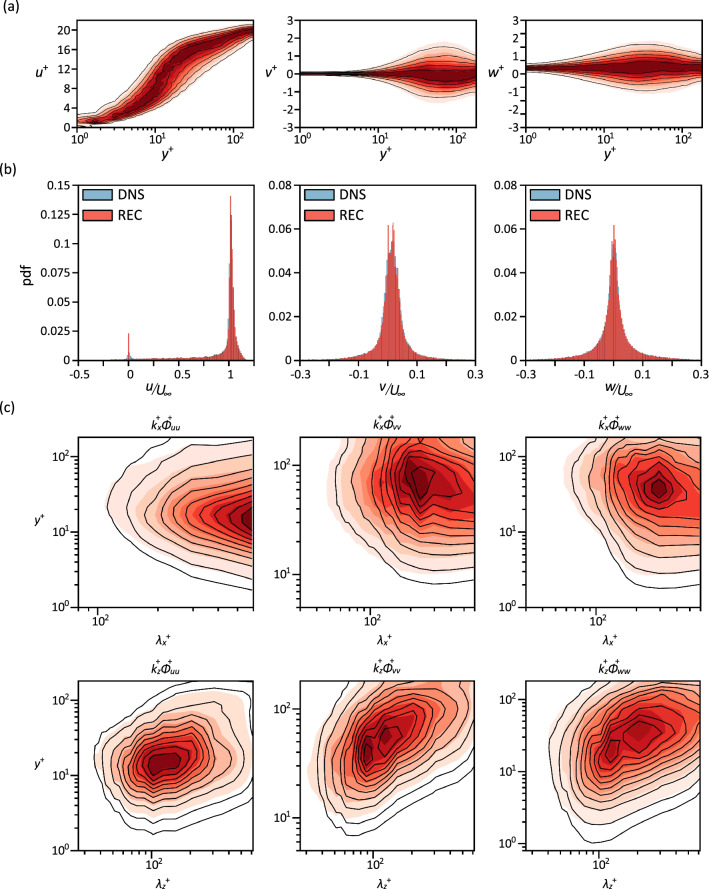
Probability density functions of the velocity components. (**a**) pdf of the turbulent channel flow case as a function of $y^+$; from left to right: streamwise velocity, wall-normal velocity and spanwise velocity. The shaded contours represent the results from the DNS data and the lines represent the results from the reconstructed data. The contour levels are in the range of 20–80% of the maximum pdf with an increment of 20%. Results for the flow at $Re_\tau =$ 180. (**b**) pdf of the velocity components for the case of flow around a finite wall-mounted square cylinder; from left to right: streamwise velocity, wall-normal velocity and spanwise velocity. Note that REC denotes reconstructed field. (**c**) Premultiplied streamwise (top) and spanwise (bottom) power-spectral density of the velocity components from the turbulent channel flow case, as a function of $y^+$ and $\lambda ^+$; from left to right: streamwise velocity, wall-normal velocity and spanwise velocity. The shaded contours represent the results from the DNS data and the lines represent the results from the reconstruction. The contour levels are in the range of 10–90% of the maximum $k_x^+ \Phi _{\alpha \alpha }^+$ and $k_z^+ \Phi _{\alpha \alpha }^+$, with increments of 10%. Results for the flow at $Re_\tau =$180.

In order to examine the ability of the proposed model to reconstruct the velocity fields with accurate spatial distribution, the probability density function of each velocity component, pdf is calculated for both cases. As shown in Fig. 3a, the pdf plots of the reconstructed velocity components for the case of turbulent channel flow show a remarkable agreement with those obtained from the DNS data, for all the regions along $y^+$. Also, for the case of flow around a finite wall-mounted square cylinder (Fig. 3b), we can see that the pdf plots are consistent with the DNS data for all the three velocity components. Here, the results indicate the ability of the model to reconstruct the instantaneous velocity fields with accurate spatial distributions.

Furthermore, the ability of the model to reproduce the spectral content of the flow in the turbulent channel is investigated by utilising the premultiplied streamwise and spanwise power-spectral densities, i.e. $k_x \Phi _{\alpha \alpha }$ and $k_z \Phi _{\alpha \alpha }$, where $k_x$ and $k_z$ are the streamwise and spanwise wavenumbers, respectively, and $\Phi _{\alpha \alpha }$ represents the corresponding wavenumber spectrum of each velocity component, $\alpha$. Figure 3c shows $k_x^+ \Phi _{\alpha \alpha }^+$ and $k_z^+ \Phi _{\alpha \alpha }^+$ as a function of $y^+$ and the corresponding inner-scaled wavelengths, $\lambda _x^+$ and $\lambda _z^+$. The spectra of the reconstructed velocity components are generally in agreement with the results obtained from DNS, with a slight deviation at high-wavenumber regions. These results further support the performance of the proposed model to properly represent the spatial distribution of the velocity fields.

**Table 1:** $L_2$-norm relative error of the reconstructed velocity fields.

Case	$\epsilon (u)$	$\epsilon (v)$	$\epsilon (w)$
Turbulent channel flow $Re_{\tau }= 180$	0.041	0.577	0.471
Turbulent channel flow $Re_{\tau }= 500$	0.044	0.589	0.515
Flow around a finite wall-mounted square cylinder	0.068	0.743	0.649

### Turbulence statistics

**Figure 4:**
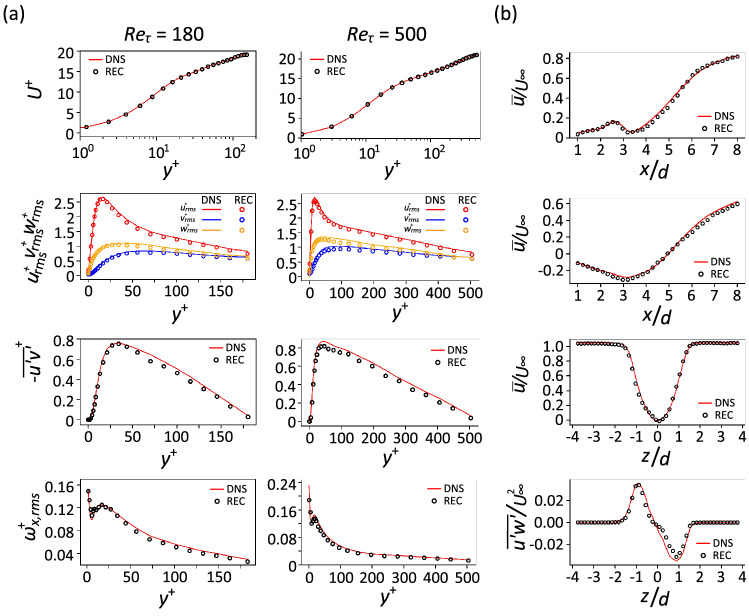
Turbulence statistics. (**a**) Turbulence statistics of the turbulent channel flow case; from top to bottom: mean streamwise velocity profile, root-mean-square profiles of the velocity components, Reynolds shear-stress stress profile and root-mean-square profile of the streamwise vorticity. (**b**) Turbulence statistics of the flow around a finite wall-mounted square cylinder case; from top to bottom: streamwise profile of mean streamwise velocity at $y/d =1~(z/d =0)$, streamwise profile of mean streamwise velocity at $y/d = 3~(z/d =0)$, spanwise profile of mean streamwise velocity at $y/d = 3~(x/d =5)$ and spanwise profile of Reynolds stress at $y/d =3~(x/d =5)$. Note that REC denotes reconstructed field.

To examine the capability of the proposed model to reproduce the turbulence statistics, the first and second-order turbulence statistics of the reconstructed velocity fields are calculated and shown in Fig. 4. As can be observed in Fig. 4a, for the case of turbulent channel flow at $Re_\tau = 180$, the profile of the mean streamwise velocity $(U^+ )$ and the root-mean-square (rms) profiles of the velocity components $(u_{\textrm{rms}}^+, v_{\textrm{rms}}^+, w_{\textrm{rms}}^+)$ are in excellent agreement with the results obtained from DNS. The Reynolds stress profile $(-\overline{u'v'}^+)$ exhibits a relatively good agreement with the results obtained from DNS, with a slight deviation in the range between $y^+$ = 50 and 125. The fluctuating streamwise vorticity $(\omega _{x, \textrm{rms}}^+)$ profile is also in agreement with the results obtained from DNS. Here the results indicate that the model can reproduce the turbulence statistics of the flow with remarkable accuracy. The turbulence statistics for the flow at $Re_\tau = 500$ indicate that the reconstructed data are generally in good agreement with the DNS. Nonetheless, the Reynolds shear-stress profile exhibits a slight underprediction for a region that starts from the maximum shear stress and continues along $y^+$. As can be seen in Fig. 4b, for the case of the flow around a wall-mounted square cylinder, the streamwise, as well as the spanwise profile of the mean streamwise velocity $({\overline{u}}/U_\infty )$ exhibit an excellent agreement with the results obtained from the DNS for the examined elevations along the cylinder height. The comparison of the spanwise profile of the Reynolds shear stress $(\overline{u'w'}/{U_\infty ^2})$ also reveals generally good agreement with the results obtained from the DNS. However, it exhibits a slight deviation at the examined wall-normal location, *i*.*e*. $y/d = 3$. This might be attributed to the increase in the complexity of the flow as the wall-normal distance increases^54,57–59^.

## Discussion and conclusions

This study presents a deep-learning-based method to reconstruct 3D turbulent flows from 2D data. A combination of 2D and 3D CNNs was utilised to build a model based on GAN, named 2D3DGAN. The model is designed to reconstruct 3D turbulent flows from a cross-plane of unpaired data (the data of each plane in the cross-plane is collected at a different time interval), such that it can be utilised to reconstruct 3D turbulent velocity fields from two-dimensional PIV measurements.

The reconstructed instantaneous velocity fields and the 3D flow structures show an excellent agreement with the DNS results for both the cases used to test the model, *i*.*e*. turbulent channel flow and the flow around a finite wall-mounted square cylinder, even for the regions where the data are not introduced to the model during the training process. The error analysis reveals that the model is robust to increasing Reynolds number and the complexity of the flow. The model also successfully reproduces the turbulence statistics with very good accuracy. Furthermore, the flow spectra for the case of turbulent channel flow also reveal a commendable agreement with the result obtained from the DNS data, indicating a great ability of the model to maintain a realistic spatial behaviour of the velocity fields.

This study demonstrates for the first time that GAN-based models can be successfully used for reconstructing 3D turbulent flows from 2D data. This approach opens the door to discovering new data-driven methods that can reconstruct the 3D turbulent flows from 2D experimental measurements and can be extended to discover more features of the model such as reconstructing 3D flow fields from 2D data for various geometries and Reynolds numbers. This would not only result in a remarkable reduction in the complexity and the cost of the experimental setup required to achieve accurate 3D experimental measurements but also in significant savings in terms of storage.

## Methods

### Building the 2D3DGAN architecture

**Figure 5:**
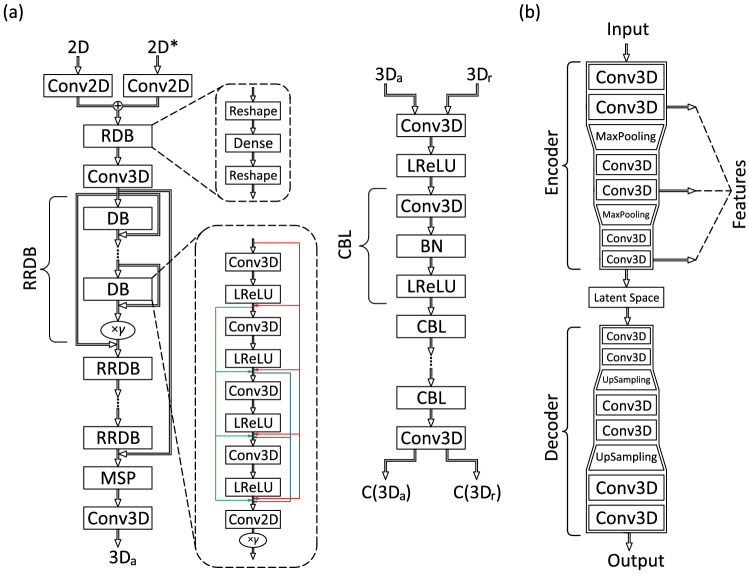
Architecture of 2D3DGAN and 3DCAE. (**a**) Schematic of 2D3DGAN showing the generator network (left) and the discriminator network (right). The generator network receives a dataset represented by two planes in the flow, the first one is the observation plane, and the second one is the plane that is matched with the observation plane by applying the procedure explained in Eq. (2), which is indicated by the superscript ‘$*$’. The output from the generator network, *i*.*e*. the artificial 3D flow data is fed to the discriminator network, and the latter tries to distinguish if the data is artificial or true. In the generator network, $\gamma$ represents the residual scaling parameter, which is set to 0.2. More information can be found in^29,43^. (**b**) Schematic of the feature extractor (3DCAE). The main features of the flow fields are extracted using three layers in the encoder part of the 3DCAE.

The architecture of 2D3DGAN is shown in Fig. 5a. Here, *G* consists of two stages. First, two sections of 2D data are introduced to *G* and passed through a 2D CNN and two stages of reshaping with a dense layer between them. After that, the 3D data are passed through a deep 3D CNN represented by residual in residual dense blocks (RRDBs) and multi-scale part (MSP). The MSP, which consists of three parallel 3D convolutional sub-models with different kernel sizes, is applied to the data features extracted by the RRDBs. More details regarding MSP can be found in Yousif et al.^29,30^. The outputs of the three sub-models are summed and passed through a final 3D convolutional layer to generate an artificial 3D data ($\mathrm{3D_a}$). The artificial and real data are fed to *D* and passed through a series of 3D convolutional, batch normalization, and leaky-ReLU layers. As a final step, the data are passed through a 3D convolutional layer. The non-transformed discriminator outputs using the real and artificial data, *i*.*e*. $\mathrm{C(3D_r )}$ and $\mathrm{C(3D_a )}$, are used to calculate the relativistic average discriminator value ($D_{Ra}$)^60^:

4 $$\begin{aligned} D_{Ra} (\mathrm{3D_r}, \mathrm{3D_a} )= & {} \sigma (\mathrm{C \left( 3D_r ) \right)} - {\mathbb {E}}_{\mathrm{3D_a}} \left[\mathrm {C ( 3D_a )} \right] , \end{aligned}$$

5 $$\begin{aligned} D_{Ra} (\mathrm{3D_a}, \mathrm{3D_r} )= & {} \sigma (\mathrm{C \left( 3D_a) \right)} - {\mathbb {E}}_{\mathrm{3D_r}} \left[ \mathrm{C (3D_r )} \right] , \end{aligned}$$

where $\sigma$ is the sigmoid function. In Eqs. (4) and  (5), $D_{Ra}$ represents the probability that the output from *D* using the real 3D data is relatively more realistic than the output using the generated 3D data.

The discriminator loss is then defined as:

6 $$\begin{aligned} L_D^{Ra} = -{\mathbb {E}}_{\mathrm{3D_r}} \left[ {\textrm{log}} (D_{Ra} (\mathrm{3D_r},\mathrm{3D_a})) \right] - {\mathbb {E}}_{\mathrm{3D_a}} \left[ {\textrm{log}} (1 - D_{Ra} (\mathrm{3D_a},\mathrm{ 3D_r })) \right] . \end{aligned}$$

The adversarial loss for the generator can be expressed in a symmetrical form as:

7 $$\begin{aligned} L_G^{Ra} = -{\mathbb {E}}_{\mathrm{3D_r}} \left[ {\textrm{log}} (1 - D_{Ra} (\mathrm{3D_r}, \mathrm{3D_a} )) \right] - {\mathbb {E}}_{\mathrm{3D_a}} \left[ {\textrm{log}} (D_{Ra} (\mathrm{3D_a}, \mathrm{3D_r})) \right] . \end{aligned}$$

The total loss function of *G* is defined as:

8 $$\begin{aligned} {\mathcal {L}}_G = \beta _1 L_G^{Ra} + \beta _2 L_{\textrm{voxel}} + \beta _3 L_{\textrm{perceptual}} + L_{\textrm{continuity}} + L_{\textrm{momentum}}, \end{aligned}$$

where $L_{\textrm{voxel}}$ is the error calculated based on the voxel (volume pixel) difference between the generated data and the ground truth data. $L_{\textrm{perceptual}}$ represents the difference in the extracted features of the real and the generated data. In this study, we use a 3D convolutional auto-encoder (3DCAE) to extract the features from the data obtained from the generator and the ground truth data as shown in Fig. 5b. Note that $L_{\textrm{continuity}}$ and $L_{\textrm{momentum}}$ represent the error of the continuity and momentum equations. In the loss function, $\beta _1$, $\beta _2$ and $\beta _3$ are the coefficients used to balance the loss terms, and their values are set to 10, 1000 and 2000, respectively. The square of the $L_2$ norm error is used to calculate all the loss terms of the generator except $L_G^{Ra}$, such that:

9 $$\begin{aligned} L_{\textrm{voxel}}= & {} \frac{1}{M} \sum _{m=1}^M \left\| \mathrm{3D_r} - \mathrm{3D_a} \right\| _2^2, \end{aligned}$$

10 $$\begin{aligned} L_{\textrm{perceptual}}= & {} \frac{1}{M} \sum _{m=1}^M \left\| {\mathscr {F}}_{FE} (\mathrm{3D_r} )- {\mathscr {F}}_{FE} (\mathrm{3D_a} ) \right\| _2^2, \end{aligned}$$

11 $$\begin{aligned} L_{\textrm{continuity}}= & {} \frac{1}{M} \sum _{m=1}^M \left\| \left( \frac{\partial u_r }{\partial x} + \frac{\partial v_r }{\partial y} + \frac{\partial w_r }{\partial z} \right) - \left( \frac{\partial u_a }{\partial x} + \frac{\partial v_a }{\partial y} + \frac{\partial w_a }{\partial z} \right) \right\| _2^2, \end{aligned}$$

12 $$\begin{aligned} L_{\textrm{momentum}}= & {} L_{\textrm{momentum}}^u + L_{\textrm{momentum}}^v + L_{\textrm{momentum}}^w, \end{aligned}$$

13 $$\begin{aligned} L_{\textrm{momentum}}^u= & {} \frac{1}{M} \sum _{m=1}^M \left\| \left( \frac{\partial u_r }{\partial t} + u_r \frac{\partial u_r }{\partial x} + v_r \frac{\partial u_r }{\partial y} + w_r \frac{\partial u_r }{\partial z} \right) - \left( \frac{\partial u_a }{\partial t} + u_a \frac{\partial u_a }{\partial x} + v_a \frac{\partial u_a }{\partial y} + w_a \frac{\partial u_a }{\partial z} \right) \right\| _2^2, \end{aligned}$$

14 $$\begin{aligned} L_{\textrm{momentum}}^v= & {} \frac{1}{M} \sum _{m=1}^M \left\| \left( \frac{\partial v_r }{\partial t} + u_r \frac{\partial v_r }{\partial x} + v_r \frac{\partial v_r }{\partial y} + w_r \frac{\partial v_r }{\partial z} \right) - \left( \frac{\partial v_a }{\partial t} + u_a \frac{\partial v_a }{\partial x} + v_a \frac{\partial v_a }{\partial y} + w_a \frac{\partial v_a }{\partial z} \right) \right\| _2^2, \end{aligned}$$

15 $$\begin{aligned} L_{\textrm{momentum}}^w= & {} \frac{1}{M} \sum _{m=1}^M \left\| \left( \frac{\partial w_r }{\partial t} + u_r \frac{\partial w_r }{\partial x} + v_r \frac{\partial w_r }{\partial y} + w_r \frac{\partial w_r }{\partial z} \right) - \left( \frac{\partial w_a }{\partial t} + u_a \frac{\partial w_a }{\partial x} + v_a \frac{\partial w_a }{\partial y} + w_a \frac{\partial w_a }{\partial z} \right) \right\| _2^2, \end{aligned}$$

where *M* represents the number of snapshots in the training mini-batch, which is fixed in this study to 8, and ${\mathscr {F}}_{FE}$ in Eq. (10) represents the function of the feature extractor, i.e. the 3DCAE.

### Overview of the training setup

The 2D3DGAN is trained separately with the data of turbulent channel flow at $Re_\tau = u_\tau \delta /\nu = 180$ and 500, and with the data of flow around a finite wall-mounted square cylinder with $AR =$ 4, and at $Re_d = U_{\infty}d /\nu = 500$, where, $\delta$ is the channel half-width. For the case of turbulent channel flow, the transfer-learning technique^16,22,29^ is used to train the model for the flow at $Re_\tau = 500$, in order to further reduce the computational cost (represented by the training time) and the required training data. For both cases, 80% of the collected data are used as training set and the rest, i.e. 20%, are used as testing set. The open-source library TensorFlow 2.4.0^61^ is used for the implementation of the 2D3DGAN. The adaptive moment estimation (Adam) optimization algorithm^62^ is used to update the weights of the model. The learning rate is initially set to $10^{-4}$ and progressively decreased during the training process to finally reach $1.25 \times 10^{-5}$. The number of trainable parameters in the model is approximately 5.7 million for the generator network and 3.74 million for the discriminator. The training of the model using a machine with a single NVIDIA TITAN RTX GPU for the case of turbulent channel flow requires approximately 48 h for $Re_\tau = 180$ and for $Re_\tau = 500$, with the aid of transfer learning, requires approximately 30 h. The training of the case of flow around a wall-mounted square cylinder requires approximately 40 h.

### Direct numerical simulation of turbulent channel flow

DNS calculations of a fully-developed incompressible turbulent channel flow at friction Reynolds numbers $Re_\tau = 180$ and 500 are performed to generate the training and testing datasets. The incompressible Navier–stokes equations are solved using the LISO code^48^, similar to the one described by Lluesma-Rodriguez et al.^63^. This code has successfully been employed to run some of the largest simulations of wall-bounded turbulent flows^52,64,65^. Briefly, the code uses the same strategy as that described by Kim et al.^66^, but uses a seven-point compact-finite difference scheme in the *y* direction with fourth-order consistency and extended spectral-like resolution^67^. The temporal discretization is a third-order semi-implicit Runge-Kutta scheme^68^. The wall-normal grid spacing is adjusted to keep the resolution to $\Delta y = 1.5 \eta$, *i*.*e*. approximately constant in terms of the local isotropic Kolmogorov scale $\eta = \left( \nu ^3 / \varepsilon \right) ^{0.25}$, where $\varepsilon$ is the isotropic dissipation of turbulent kinetic energy.

The dimensions of the computational domain for both simulations are set to $8 \pi \delta , 2 \delta$ and $3 \pi \delta$ in the streamwise, wall-normal and spanwise directions, respectively. The total number of grid points is around 50 million for the flow at $Re_\tau =$ 180 and 444 million for the flow at $Re_\tau =$ 500. A uniform distribution of the grid points is used in the streamwise and spanwise directions and a non-uniform distribution is used in the non-homogeneous wall-normal direction. For the flow at $Re_\tau = 180$, the grid spacings in the streamwise ($\Delta x^+$) and spanwise directions ($\Delta z^+$) are 8.55 and 4.27, respectively, while for the flow at $Re_\tau =$ 500, $\Delta x^+ = 8.33$ and $\Delta z^+ = 4.16$. The grid spacing near the wall in the wall-normal direction is $\Delta y_w^+ = 0.53$ and 0.74 for the flow at $Re_\tau =$ 180 and 500, respectively. The simulation time step ($\Delta t^+$) is set to 0.07 and 0.09 for the flow at $Re_\tau =$ 180 and 500, respectively. The flow is periodic in the streamwise and spanwise directions, whereas the no-slip condition is applied to the channel walls. A total of 1000 consecutive snapshots are collected for each of the two simulations.

### Direct numerical simulation of flow around a finite wall-mounted square cylinder

In the second case, we consider the flow around a finite wall-mounted square cylinder with $AR =$ 4, at $Re_d = 500$. The spectral-element-method (SEM)-based open-source code Nek5000 developed by Fischer et al.^55^ is used to perform the DNS. In the SEM^69^, the computational domain is decomposed into elements, and the solution is expressed in terms of Lagrange interpolants of order *N* within those elements. The location of the nodes inside the elements follows the Gauss–Lobatto–Legendre (GLL) distribution, whereas there is an isoparametric mapping for the shape of the elements and there are no restrictions regarding the position of the elements in the domain. This means that this method allows the flexibility to compute complex geometries, while still preserving the characteristics of a high-order spectral method. In the present study, the velocity field is expressed in terms of Lagrange interpolants of order $N=5$, and order $N-2=3$ is considered for the pressure field. The nonlinear terms are treated explicitly by third-order extrapolation (EXT3), whereas the viscous terms are treated implicitly by a third-order backward differentiation scheme (BDF3). The no-slip boundary condition is applied for the cylinder walls and the ground, while periodic boundary conditions are used in the spanwise direction. At the top of the domain, we impose a constant streamwise velocity, a zero spanwise velocity and zero stress in *y*. Furthermore, the input is a laminar boundary layer, and the output is the stabilized boundary condition by Dong et al.^70^. The dimensions of the simulation domain are $( L_x, L_y, L_z )d = (60, 12, 12)$ in *x*, *y* and *z*, respectively, and the total number of grid points is around 20 million. We collect a total of 10,000 snapshots with a time setp among snapshots of $\Delta t U_\infty / d = 0.02$.

### Data preparation and pre-processing

To reduce the computational cost of training the model and to increase the training and testing data, each simulation domain in the case of turbulent channel flow is divided into 12 identical sub-domains having a size of $2 \pi \delta$, $2 \delta$ and $\pi \delta$. Furthermore, the grid size of each sub-domain is interpolated and reduced to $64 \times 48 \times 48$ grid points with a uniform distribution in the *x* and *z* directions and a non-uniform distribution in the *y* direction. For the case of flow around a wall-mounted square cylinder, the size of the domain that is used for training and testing the model is set to $10d \times 7.6d \times 8d$, and the data are interpolated and reduced into a uniform grid distribution of $48 \times 48 \times 48$ points. The input data to the model are normalised using the min-max normalisation to obtain values between 0 and 1.

## Data Availability

All the data analysed in this paper were produced with the in-house and open-source softwares described in the code-availability statement. Reference data and the scripts used to produce the data figures, as well as instructions to train and test the 2D3DGAN are available on the following web page: https://fluids.pusan.ac.kr/fluids/65416/subview.do.

## Code availability

The DNS calculations of the turbulent channel flow case are performed using in-house code LISO, contact SH for the code availability. The DNS calculations of the case of flow around a finite wall-mounted square cylinder are performed using the open-source code Nek5000 (https://nek5000.mcs.anl.gov/). The open-source library TensorFlow 2.4.0 is used for the implementation of the 2D3DGAN (https://www.tensorflow.org/). The source code of the presented model is available on the following web page: https://fluids.pusan.ac.kr/fluids/65416/subview.do.
